# Catchment area characteristics do not account for geographical variation in ADHD diagnoses

**DOI:** 10.1007/s00787-025-02720-x

**Published:** 2025-04-24

**Authors:** Tore Hofstad, Olav Nyttingnes, Ingvar Bjelland, Arnstein Mykletun

**Affiliations:** 1 Centre for Population Health, Haukeland University Hospital, Bergen, Norway; 2 Centre for Medical Ethics, University of Oslo, Oslo, Norway; 3 Health Services Research Unit, Akershus University Hospital, Lørenskog, Norway; 4 Department of Clinical Medicine, University of Bergen, Bergen, Norway; 5 Division of Psychiatry, Haukeland University Hospital, Bergen, Norway; 6 Centre for Work and Mental Health, Nordland Hospital Trust, Bodø, Norway; 7 UiT—The Arctic University of Norway, Tromsø, Norway; 8 Division for Health Services, Norwegian Institute of Public Health, Oslo, Norway

**Keywords:** Attention-Deficit/Hyperactivity disorder (ADHD), Geographical variation, Service use, Provider preference, Social inequality

## Abstract

**Background:**

Geographical variation in Attention-Deficit/Hyperactivity Disorder (ADHD) diagnoses remains poorly understood. Previous research has found that the variation in ADHD rates between Child and Adolescent Mental Health Services (CAMHS) is not attributable to ADHD symptom load in the catchment areas of the CAMHS. This study aimed to investigate if geographical variation in rates of ADHD-diagnosis per population and per patient, as well as referral rates to CAMHS, were associated with catchment area characteristics.

**Methods:**

We used data from the Norwegian Patient Registry, covering everyone aged 5–18 in contact with CAMHS 2009–2011, and catchment area level data from Statistics Norway, including population size, centrality, socioeconomic position, welfare reliance, and general population health. Spearman’s Rho was calculated to assess the strength and direction of monotonic correlation.

**Results:**

Referral rates to CAMHS per population were lower in catchment areas with higher proportion of non-European population; more urban areas; fewer adults receiving disability benefits; and more economic inequality. Higher rates of ADHD per population were seen in areas characterised by lower socio-economic position. The rate of ADHD diagnosis per patient in CAMHS ranged from 5 to 27% and was not associated with catchment area characteristics.

**Conclusion:**

This study found that the proportion of ADHD diagnoses per population was moderately related to catchment area characteristics, following a social gradient in health. However, the proportion of ADHD diagnoses among patients in CAMHS was unrelated to area characteristics. This suggests that other factors, such as variation in clinician attitudes or local practice styles, may contribute to the strong variation in ADHD diagnoses.

**Supplementary Information:**

The online version contains supplementary material available at 10.1007/s00787-025-02720-x.

## Introduction

Geographical variation in ADHD diagnosis has been documented in several countries where residents are expected to have relatively equal access to health services [1–3], including the UK, Denmark and Norway [4]. To explain these variations, four hypotheses, which are not mutually exclusive, can be proposed:

### H1

*Morbidity*: The variation in ADHD diagnoses across geographical regions reflects actual differences in the level of ADHD symptoms in the population.

### H2

*Healthcare Access and Capacity*: The variation in ADHD diagnoses could be caused by variation in the capacity of and access to mental health services across regions.

### H3

*Socio-Economic Inequality*: Socioeconomic disparities between CAMHS catchment areas may also contribute to the variation in ADHD diagnoses established in CAMHS through *H3a: Social Gradient in Health* impacting morbidity, and *H3b: Health Seeking Behaviour*, which impacts likelihood of healthcare access.

### H4

*Attitudes*: The variation in ADHD diagnoses is caused by variation in attitudes among clinical staff responsible for diagnostic assessment and treatment (psychiatrists, psychologists) at the different CAMHS.

Geographical variations in ADHD diagnoses within a country can be problematic if they reflect inconsistencies in diagnostic practices rather than true differences in prevalence [5], because it indicates unequal access to health care for the population. We recently tested and rejected the morbidity hypothesis and found that the variation in ADHD diagnoses per population between CAMHS was largely unrelated to variation in ADHD symptom levels in the general population of their catchment areas [4].

The healthcare access and capacity hypothesis can be derived from findings of Wennberg et al. [6], which suggest that increased referrals and subsequent consultations provide more opportunities for diagnoses and tend to result in a higher number of diagnoses. Wennberg calls this type of variation supply-sensitive, and argues it is *unwarranted* because it implies that medical practice is influenced more by local practice styles and the availability of resources rather than by evidence-based medicine or patient needs. In Denmark, Madsen et al. [2] found support for this hypothesis with regards to ADHD, and in the United States, Bruckner et al. [7] found more prescriptions in areas with more physicians, which they suggested could be related to higher diagnostic rates of ADHD.

The socio-economic inequality hypothesis can be derived from a substantial body of evidence showing that children from households of lower socio-economic position have a higher risk of mental illness [8], including ADHD [9]. This is one of the reasons why CAMHS capacity in Norway is partly dimensioned by catchment area characteristics such as morbidity and social deprivation [10]. In the United Kingdom, Prasad et al. [11] found higher levels of ADHD-diagnoses in more disadvantaged areas, while in Denmark, Madsen et al. [2] found no support for the Socio-economic inequality hypothesis.

Lyhman et al. [12] investigated the attitude hypothesis and found that attitudes among clinicians at CAMHS exist on a continuum ranging from «liberal» to «restrictive» towards providing ADHD diagnoses. «Liberal» clinicians tend to believe that diagnosis and treatment are beneficial for children and adolescents with symptoms of ADHD, thus having a lower threshold for providing a diagnosis. In contrast, «restrictive» clinicians are concerned about medicalisation and the stigma following diagnosis, preferring a strategy of watchful waiting. Consequently, they place the threshold for diagnosis higher on the symptom scale.

The aim of this study is to examine the second and third hypotheses, specifically to estimate associations between rates of ADHD diagnosis and characteristics of CAMHS catchment areas. These characteristics may influence both the demand for and supply of CAMHS services, which will be reflected in referral patterns, diagnostic practices, and treatment options.

Our research questions are as follows:

What is the direction and strength of the correlation between CAMHS catchment area characteristics and:

1. The proportion of patients in contact with CAMHS per population.
2. The proportion of persons diagnosed with ADHD per population.
3. The proportion of persons diagnosed with ADHD per patient in CAMHS.

## Methods

### Data sources and variables

Individual level data for everyone aged five to eighteen in contact with CAMHS between February 2009 and January 2012 (*N* = 81 150) was obtained from the Norwegian Patient Registry. The *proportion of patients in contact with CAMHS per population* was calculated by dividing the number of persons in contact with specialist mental health services with the population aged five to eighteen in the catchment area. Persons who moved between catchment areas during the study period were counted in each area for this variable. *The proportion of persons diagnosed with ADHD per population* was calculated by dividing the number of persons receiving an ADHD diagnosis within two years of first contact with CAMHS by the area’s population-at-risk. *The proportion of persons diagnosed with ADHD per patient in CAMHS* was calculated by dividing the number of persons diagnosed with ADHD within two years of first contact with CAMHS with the number of persons in contact with this service. As a measure of practice variation in CAMHS, *the average number of contacts per patient* was calculated by counting the number of separate days with registered contacts for each patient at CAMHS and dividing by the number of patients.

The following variables on municipality and city district characteristics were obtained from Statistics Norway: population aged five to eighteen; centrality index, where a high value means more urban; proportion of children living in low-income households; median income; Gini-coefficient; upper secondary school drop-out rate; employment rate; and the proportion of inhabitants receiving lifetime disability benefits. Life expectancy data at the municipal level were obtained from the Norwegian Institute of Public Health.

The variables were aggregated from municipal level to catchment area to CAMHS by calculating the population-weighted average. *The proportion of the population aged five to eighteen with a non-European background* was calculated using a control cohort matched on age and gender, with no contact with CAMHS during the study period (*N* = 95 882), by dividing the number of individuals born outside Europe by the population aged five to eighteen in the area. The supplementary online resources contain a figure which shows correlations between the catchment area characteristics.

### Theoretical rationale for selection of area indicators

The population aged five to eighteen is the primary group at risk of receiving ADHD diagnosis and treatment, and its *catchment area size* can influence both the demand for CAMHS services and the incidence of ADHD [10, 13]. Ethnic and cultural factors, particularly in *non-European populations*, may lead to different ADHD diagnosis rates, influenced by mental health perceptions, stigma, healthcare utilisation, as well as socio-economic position [14]. Urban and rural areas, measured by the *centrality* index, may differ in healthcare accessibility, environmental and lifestyle factors which can impact CAMHS contact and ADHD incidence, with urban regions potentially offering better healthcare access, but also greater exposure to environmental risk factors [15].

Given the challenges in measuring the socio-economic position of residents, we use a wide range of indicators. *Life expectancy* reflects overall health and socio-economic conditions, potentially correlating with healthcare quality and ADHD diagnosis rates [9]. Similarly, *median income* is linked to healthcare access and the likelihood of ADHD treatment seeking. *Children in low-income housing* are subject to stressors and environmental influences that may exacerbate mental health issues, including ADHD. Income inequality, measured by the *Gini coefficient*, could affect ADHD diagnosis and service utilization due to social stressors and healthcare access disparities [16]. *Upper secondary school dropout rates* signify community-wide social and educational challenges, possibly creating both barriers to, and need for, mental health services [17, 18]. Lastly, *employment rates*, as well as the inverse measure *the proportion of the population receiving lifetime disability benefits* signal the community’s overall functioning and health burden, encompassing mental health disorders [10].

### Study setting

Norway is a suitable setting for this study, as it has a uniform diagnostic standard, a universally available health care system which is free for children, and a high-quality registry detailing all contacts with CAMHS.

### Missing data

There were some missing values in the municipality level data: disability benefits (4, 1%); upper secondary school dropout (30, 6.6%); Gini (2, 0.4%); median income (3, 0.7%); children in low income housing (58, 13%); work assessment allowance (3, 0.7%); life expectancy (103, 23%). The R-package mice 3.15.0 (22) was used to perform multiple imputation by chained equations, using predictive mean matching with 50 imputations. All area characteristics except life expectancy; the number of children in low-income housing and the proportion of the population receiving work assessment allowance and disability benefits were included in the imputation model.

### Statistical analysis

The strength of monotonic correlations was calculated using Spearman’s Rank Order Coefficient (ρ). Correlation coefficients were interpreted using conventional thresholds: < 0.30 as small, 0.30–0.50 as moderate, and > 0.50 as strong correlations. P-values were adjusted for multiple testing using the Holm-Bonferroni correction [19], while 95% confidence intervals were based on unadjusted standard errors. All parameters were pooled using Rubin’s rules (20), using the with() function from mice. All analyses were performed in R version 4.2.2 [20]. Correlations were calculated using the correlation package version 0.8.3 [21]. Models with alternative strategies to account for differences in population size can be found in the supplementary files.

## Results

Table 1 shows the distribution of characteristics of catchment areas to CAMHS.

**Table 1:** Distribution of characteristics of catchment areas to CAMHS in Norway 2011. Data from statistics Norway and the Norwegian Institute of public health were aggregated from municipality/city district and weighted by the area’s at-risk-population aged 5–18

CAMHS characteristics	Minimum	Maximum	Mean	Standard deviation
% Patients at CAMHS per population*	6	17	10.6	2.3
% ADHD per population*	0.5	4.3	1.5	0.7
% ADHD per patient in CAMHS*	5	27	14.4	5.4
Average contacts per patient	11	18.3	14.5	1.7
Size of catchment area	2 K	54 K	12 K	10 K
% Non-European population aged 5 to 18	3	66	13.3	12
Centrality (high = more urban)	517	1 K	764	136
Life expectancy	78.9	83	81.3	0.8
Median income	472 K	731 K	553 K	57 K
Gini (high = more unequal)	0.18	0.37	0.22	0.032
% High school dropout	13	41	26.2	5.4
% Employed	61	74	68.6	2.6
% Lifetime disability benefits	5	18	10.9	3.4

*: Three-year incidence

**Fig. 1:**
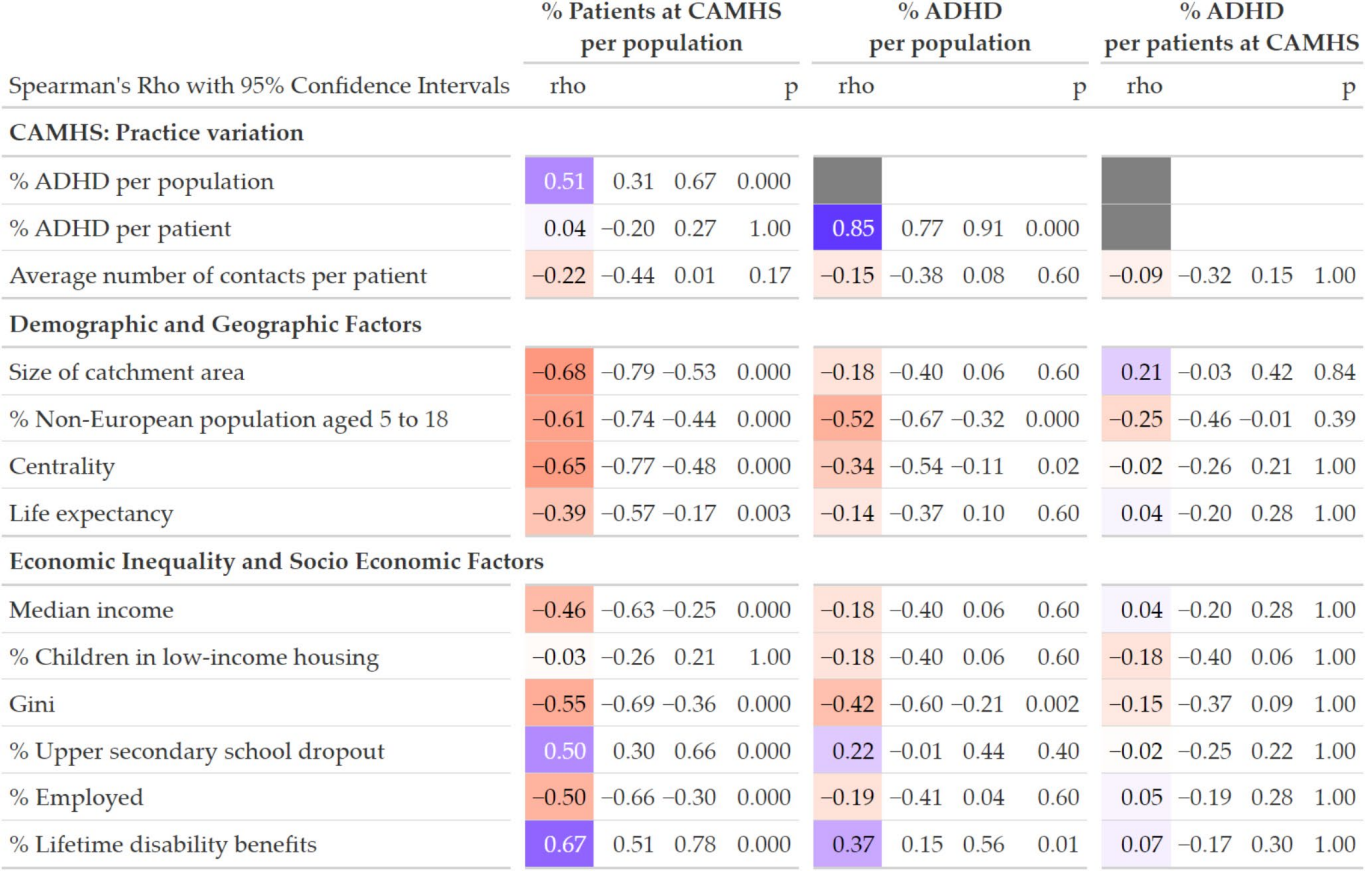
Correlations between Child and Adolescent Mental Health Service (CAMHS) practice variation and catchment area characteristics, based on aggregated data from the Norwegian Patient Registry, Statistics Norway, and The Norwegian Institute of Public Health. Correlation strength is interpreted as small (ρ < 0.30), moderate (ρ = 0.30–0.50), or strong (ρ > 0.50)

There were several moderate to strong associations between catchment area characteristics and the proportion of patients in contact with CAMHS per population in the catchment area (Fig. 1, left column). These contact rates varied from 6 to 17%, and fewer children and adolescents were in contact with CAMHS in more populated, urban areas, more prosperous and less economically unequal areas, in areas where a higher rate of children had a non-European background, in areas with lower rates of high school dropout, and in areas where fewer adults were unemployed or received welfare benefits. The rate of ADHD diagnoses per population followed a similar, but weaker pattern of associations with catchment area characteristics (Fig. 1, middle column). Models with alternative strategies for accounting for population size showed weaker associations between area characteristics and the proportion of patients in contact with CAMHS per population, but with a similar pattern of associations (see supplementary material).

Among patients referred to CAMHS, there were no statistically significant association (all *p* >.05) between ADHD diagnosis and catchment area characteristics, and all correlation coefficients ranged from small to zero (Fig. 1, right column).

## Discussion

Our analysis showed that area characteristics are related to indicators of CAMHS practice in different ways. Area characteristics are associated with mental health service use among children and adolescents, as shown by the moderate to strong correlations with the proportion of patients in contact with CAMHS, suggesting that regional factors are related to the likelihood of seeking and receiving mental health services. Many of these correlations follow a social gradient in health, with more persons in contact with CAMHS in areas with more upper secondary school dropout and unemployment; more people receiving welfare benefits; and lower median income.

Some of these area characteristics also showed small to moderate correlations with the prevalence of ADHD diagnoses per population. We infer that the underlying reason for these correlations is largely due to contact rates with CAMHS being a prerequisite for diagnosis. This inference is supported by the moderate correlation between ADHD per population and proportion of patients in contact with CAMHS (ρ = 0.51). Combined, these findings provide partial support to the Healthcare access and capacity hypothesis.

The observed correlations are consistent with the priorities of the Norwegian healthcare budgeting framework [10], which intends to adjust the CAMHS budget according to population size, with slight modifications for varying population needs. These strong correlations indicate that the targeted budget allocation may be working as intended by policymakers. However, our findings might indicate that increasing CAMHS capacity through increased budgets might also increase diagnoses rates in the population, without a corresponding increase in actual morbidity, since a previous study found that actual morbidity indicators could not predict diagnostic rates of ADHD [4]. This is in line with Wennberg et al.’s observations that more physicians in an area leads to more consultations, which tend to result in more diagnoses [6]. A possible interpretation of this finding is that the population in areas characterised by lower socio-economic position are being medicalised, with relative overcapacity resulting in overdiagnosis [22].

In notable contrast, the considerable variation in ADHD diagnoses per patient in CAMHS across catchment areas, ranging from 5 to 27%, shows little to no correlation with area characteristics. This lack of correlation indicates that upon contact with CAMHS, area characteristics do not account for ADHD diagnoses. Instead, other elements must contribute to this variability, which supports the attitude-hypothesis; implying that diagnostic rates are influenced by provider preferences or local clinical culture [12, 23].

Additionally, the proportion of non-European population in an area showed a weak negative association with the proportion of ADHD diagnoses among those in contact with CAMHS. This reflects the stronger negative association with contact rates, suggesting that non-European populations may use mental health services less frequently. Possible reasons include stigma, institutional mistrust, language barriers, or a lack of awareness of healthcare entitlements. Consequently, there is a risk of underdiagnosis of mental health conditions, including ADHD, in these populations. Conversely, this could also suggest a reduced probability of overdiagnosis in these communities.

Finally, the average number of contacts with CAMHS per patient was not significantly associated with any of our three «outcome»-variables. However, the estimates suggest that CAMHS with a higher proportion of patients per population on average spend fewer days in contact per patient.

### Strengths and limitations

The comprehensive nature of the registry data is a strength of this study. Since we had access to data for every person in contact with CAMHS during the study period, the results are representative of real world practice.

The ecological nature of the study limits our ability to make individual-level inferences. It is worth emphasising that neither of these associations ought to be interpreted causally, as they might be spurious and confounded by each other. Future studies could use multilevel or causal methods to more precisely disentangle the influence of individual and contextual factors.

The variable indicating the proportion of the population receiving lifetime disability benefits was sourced from 2015 data, beyond our study period. We included it because earlier data was not available from Statistics Norway, and we consider the impact on the estimates to be negligible, given the observed year-to-year stability of this indicator.

Registry owners required that the data was provided with pseudonymised id for geographical areas, which precluded us from making qualitative investigations and geospatial analyses.

We did not have access to data on staffing at CAMHS, which limited the possibility of thoroughly assessing the supply-sensitivity of ADHD diagnoses. We also lacked data on clinician attitudes and local diagnostic practices. Future studies could address this limitation through qualitative interviews with clinicians to explore the extent to which such factors may contribute to diagnostic variation.

An important limitation of our analysis is the potential for spurious associations arising from the use of population size in both the numerator and denominator of several variables [24]. Specifically, the associations between catchment area characteristics and both *the proportion of patients in contact with CAMHS per population* and *the proportion of persons diagnosed with ADHD per population* may be artificially inflated due to this shared denominator. To address this concern, we conducted additional regression-based analyses that account for population size, the results of which are provided in the supplementary material. Although these models showed some attenuation of the associations related to the proportion of patients in contact with CAMHS per population, the overall patterns and directions of the relationships remained consistent with the results from the more parsimonious models reported in the main analysis.

## Conclusions

The moderate to strong correlations between area characteristics and the proportion of patients at CAMHS per population suggest that these characteristics can be predictive of the likelihood of individuals engaging with mental health services. While this appears to follow policy-makers intentions of reducing inequality, it can paradoxically result in a relative overdiagnosis of ADHD, given the lack of variation in ADHD symptoms in the population. The proportion of ADHD diagnoses per population was moderately related to catchment area characteristics, following a social gradient in health. However, once individuals were in contact with CAMHS, the same area characteristics did not account for the reception of an ADHD diagnosis. So, a large share of the geographical variation in ADHD diagnoses remains unexplained. Variation in diagnostic attitudes and preferences among clinicians at CAMHS continue to be a plausible explanation for the strong variation in rate of ADHD diagnoses between CAMHS.

## Electronic supplementary material

Below is the link to the electronic supplementary material.

Supplementary Material 1

## Data Availability

Data on area characteristics are available free of charge from Statistics Norway. Data from the Norwegian Patient Registry can be acquired for a fee through formal channels but cannot be shared by the authors.

## Code availability

Relevant parts of the code can be shared at a reasonable request.

## Declarations

### Disclaimer

Data from the Norwegian Patient Registry has been used in this publication. The interpretation and reporting of these data are the sole responsibility of the authors, and no endorsement by the Norwegian Patient Registry is intended nor should be inferred.
