# Supplementary material for: Catchment area characteristics do not account for geographical variation in ADHD diagnoses
Source: Eur Child Adolesc Psychiatry. 2025 Apr 24;34(10):3097–103. doi: 10.1007/s00787-025-02720-x (PMC12592264; doi:10.1007/s00787-025-02720-x)
Supplement: Supplementary file 1 — Supplementary Material 1 [file 787_2025_2720_MOESM1_ESM.docx]

**
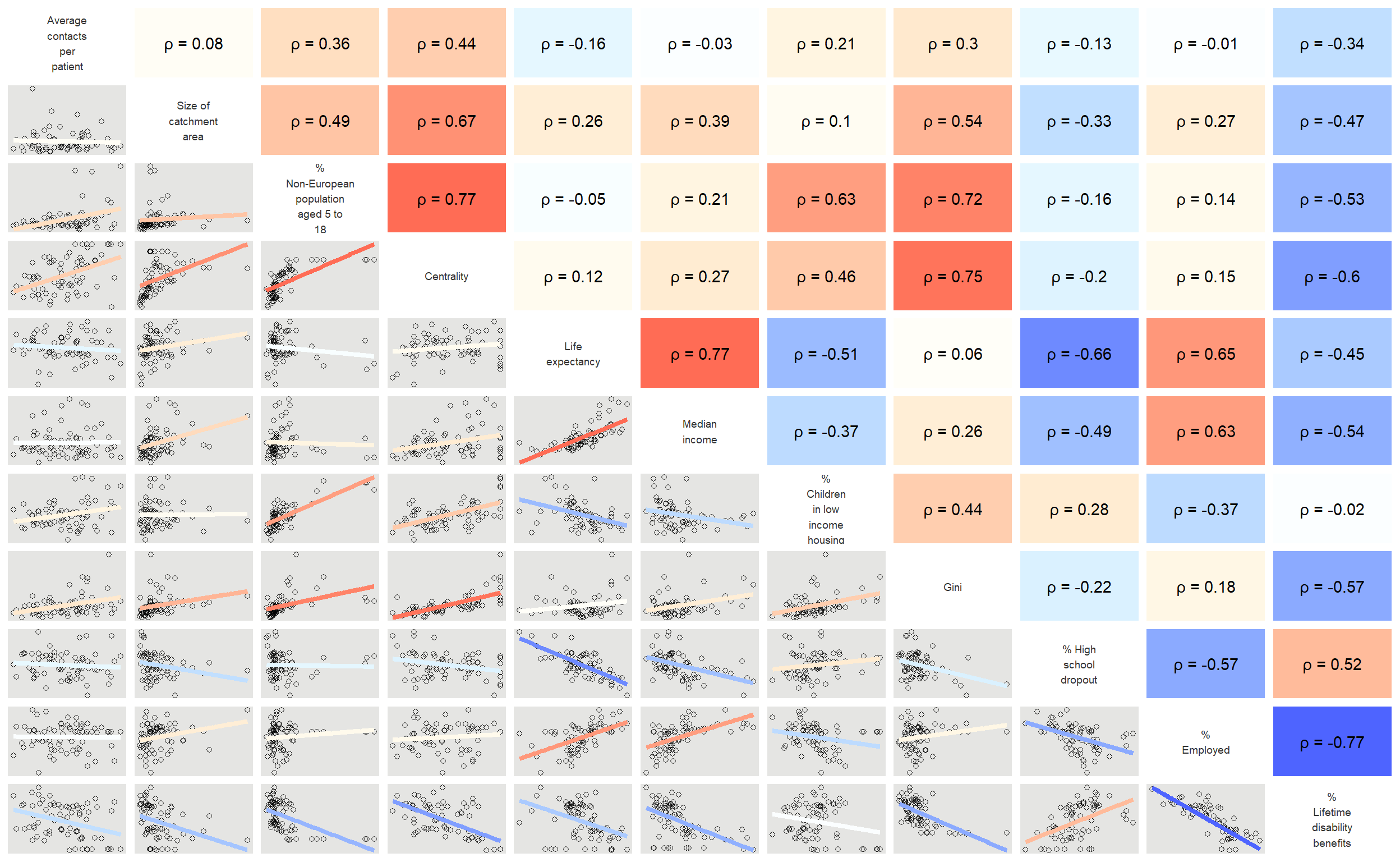
**

Online Resource 1: Correlation plot of characteristics of catchment areas to CAMHS in Norway 2011, based on aggregated data from the Norwegian Patient Registry, Statistics Norway, and The Norwegian Institute of Public Health. Heatmap with Spearman’s ρ.

*Online Resource 2: Linear regression of rank-transformed variables, with* $\frac{1}{population}$ *added as covariate. Associations between Child and Adolescent Mental Health Service (CAMHS) practice variation and catchment area characteristics, based on aggregated data from the Norwegian Patient Registry, Statistics Norway, and The Norwegian Institute of Public Health*
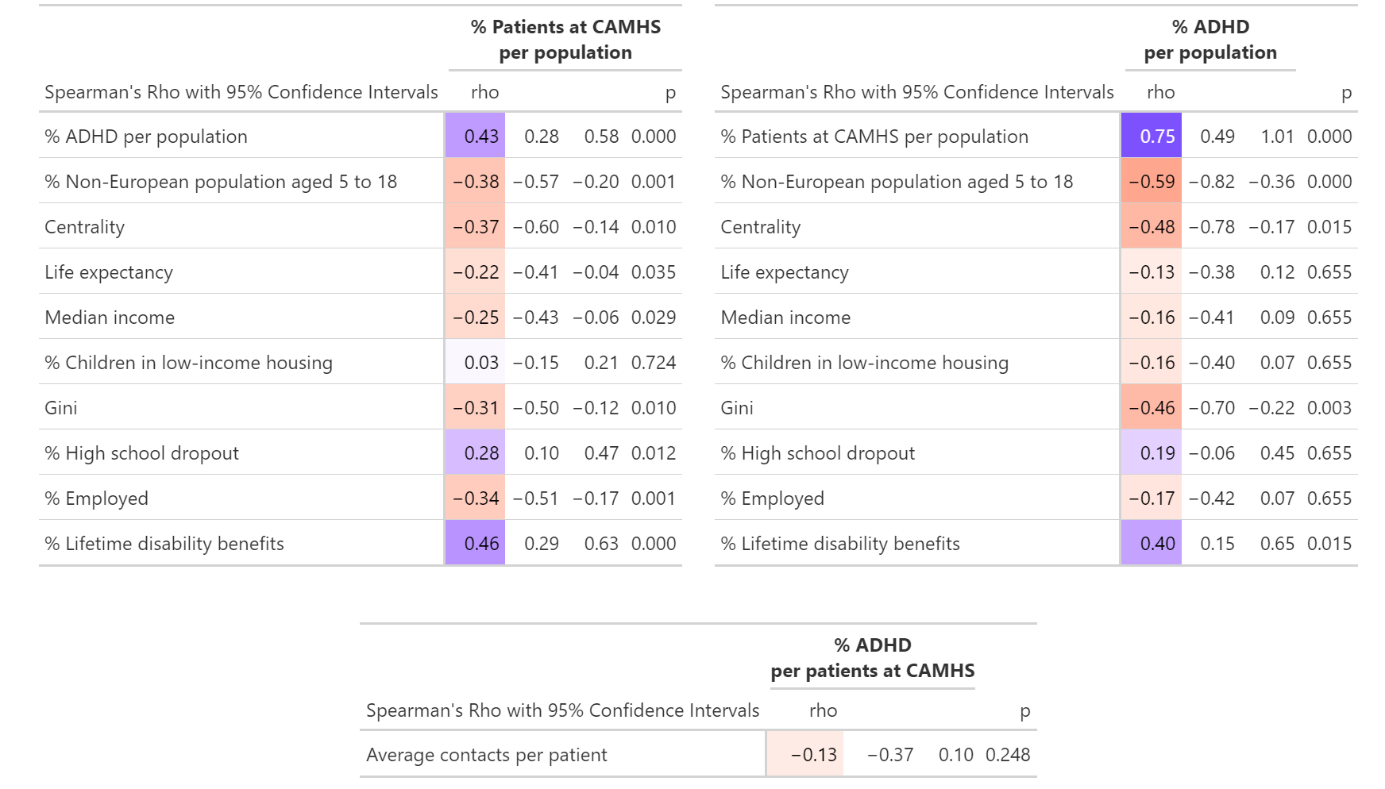
*.*

Methods: To mitigate the risk of spurious associations resulting from correlating ratios with a common denominator, we employed a regression-based approach. A ratio such as $\frac{ADHD}{population}$ can be expressed as ADHD × $\frac{1}{population}$. Accordingly, we conducted linear regressions where the inverse of the denominator ($\frac{1}{population}$) was included as a covariate [24]. For the variable «average contacts per patient in CAMHS», the inverse of the denominator ($\frac{1}{patient at CAMHS}$) was similarly added as a covariate. By applying a rank transformation to each variable, the resulting coefficients are equivalent to Spearman's rho, enabling direct comparison with coefficients from the main analysis. Similar to the main analysis, p-values were adjusted for multiple testing using the Holm-Bonferroni correction, while 95% confidence intervals were based on unadjusted standard errors.

## Reference:

Firebaugh, G., & Gibbs, J. P. (1985). User's guide to ratio variables. *American Sociological Review*, 713-722.
